# Seal finger following a photography incident

**DOI:** 10.1016/j.idcr.2021.e01098

**Published:** 2021-03-31

**Authors:** Gregorius J. Sips, Nina Bakashvili, Els M. Broens, Carolina A.M. Schurink

**Affiliations:** a Department of Medical Microbiology and Infectious Diseases, Erasmus University Medical Center, Rotterdam, The Netherlands; b Medical Centre De Stadsdriehoek, Rotterdam, The Netherlands; c Department of Biomolecular Health Science, Faculty of Veterinary Medicine, Utrecht University, Utrecht, The Netherlands; d Department of Internal Medicine, Erasmus University Medical Center, Rotterdam, The Netherlands

**Keywords:** Seal finger, Bite wound, Wildlife selfie

A 20-year-old woman consulted a general practitioner with a highly painful and swollen right middle finger following multiple bite injuries of the right hand, particularly of the middle finger (Fig. 1). She had suffered the injuries while taking a photograph during a minicruise along the coast of Scotland when a seal had jumped out of the water and bitten her hand. Given the nature of the injuries and lack of response to initial treatment with beta-lactam antimicrobials, a seal finger, also known as “spekk” or “blubber” finger was suspected [1,2]. Seal finger was first described in 1907, traditionally in seal hunters and more recently in wildlife workers [1,2]. The exact etiology was unknown for a long time, but in 1991 *Mycoplasma* species in the oral cavity of seals were linked to human infection [[1], [2], [3], [4]]. A recent microbiome analysis of the oral cavity of seals indeed demonstrated that the oral cavity of seals contains several *Mycoplasma* species, *M. phocicerebrale* (originally described as *M. phocacerebrale*) being a widespread -identifiable- species, which were also found in skin lesions [5,6]. Beta-lactam antimicrobials are recommended as empiric therapy for treatment of (animal) bite injuries but, as *Mycoplasma* species lack a peptidoglycan cell wall, these antimicrobials are ineffective [1,2]. Successful treatment with tetracyclines has been reported [[1], [2], [3]]. The patient’s lesions responded well to a 10-week course of doxycycline 200 mg daily. Treatment duration was prolonged in regard to earlier case reports (2–6 weeks [1,2]) because of a deep soft tissue wound of the right middle finger.

**Fig. 1:**
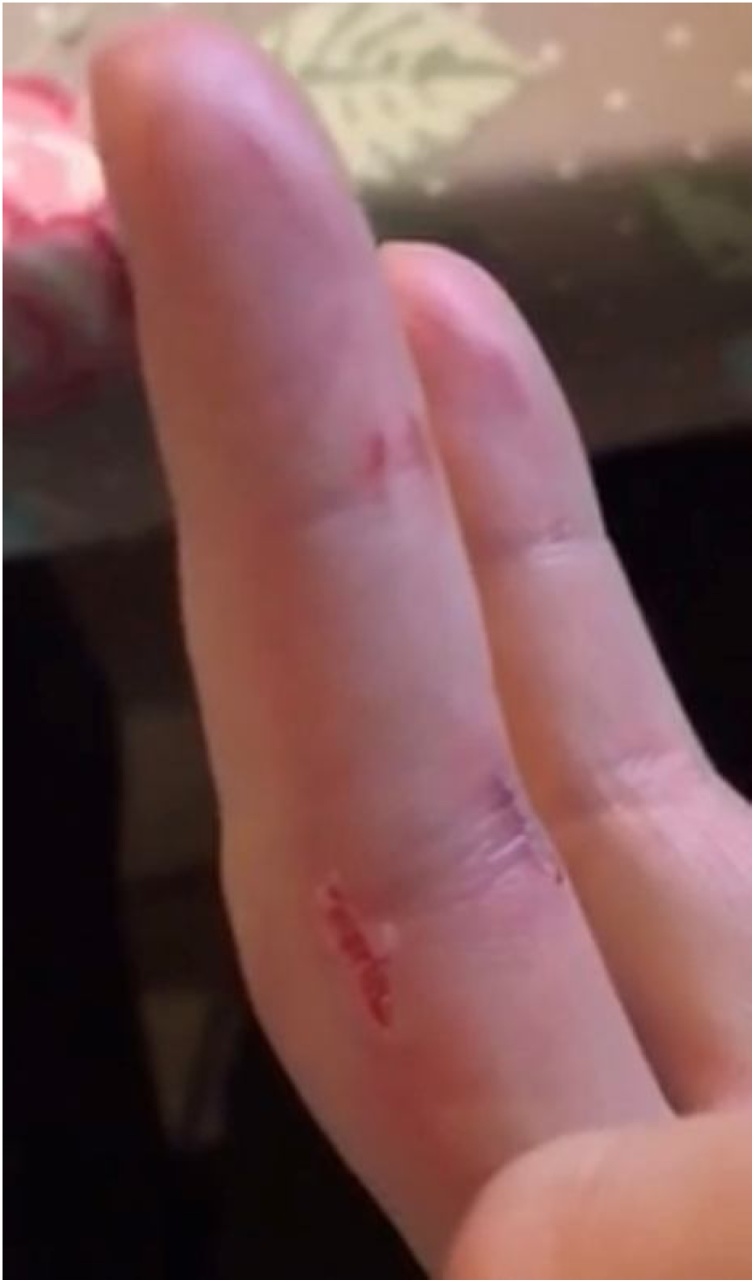
Bite injuries of the right middle finger.

Although not the case here, ‘wildlife selfies' constitute a relatively novel phenomenon that has drawn significant attention of the medical community. It is good that doctors are aware of this phenomenon and potential risks associated with capturing close ‘wildlife selfies’ [7].

## Declaration of Competing Interest

George Sips: no conflicts of interest.

Nina Bakashvili: no conflicts of interest.

Els Boens: no conflicts of interest.

Carolina Schurink: no conflicts of interest.

## Sources of funding

None.

## Ethical approval

NA.

## Consent

Written informed consent was obtained from the patient for publication of this case report and

accompanying images. A copy of the written consent is available for review by the Editor-in-Chief of this journal on request.

## Author contribution

Gregorius Sips: Writing- Original Draft, Writing-Review & Editing

Nina Bakashvili: Writing-Review & Editing

Els Broens: Writing-Review & Editing

Carolina Schurink: Writing-Review & Editing

## Contributors

GJS and CAM provided tertiary-care treatment advise. NB provided primary-care treatment advise and images. EMB provided veterinary expertise. GJS drafted the report. All authors read and approved the final manuscript.
